# Once‐a‐week or every‐other‐day urethra‐sparing prostate cancer stereotactic body radiotherapy, a randomized phase II trial: 18 months follow‐up results

**DOI:** 10.1002/cam4.2966

**Published:** 2020-03-11

**Authors:** Thomas Zilli, Sandra Jorcano, Samuel Bral, Carmen Rubio, Anna M.E. Bruynzeel, Angelo Oliveira, Ufuk Abacioglu, Heikki Minn, Zvi Symon, Raymond Miralbell

**Affiliations:** ^1^ Geneva University Hospital Geneva Switzerland; ^2^ Teknon Oncologic Institute Barcelona Spain; ^3^ Onze‐Lieve‐Vrouwziekenhuis Aalst Belgium; ^4^ Hospital Universitario HM Sanchinarro Madrid Spain; ^5^ Amsterdam UMC, location VUmc Amsterdam the Netherlands; ^6^ Portuguese Institut of Oncology Porto Portugal; ^7^ Neolife Medical Center Istanbul Turkey; ^8^ University Hospital Turku Turku Finland; ^9^ Sheba Medical Center Ramat Gan Israel

**Keywords:** overall treatment time, prostate cancer, quality of life, stereotactic body radiotherapy, urethra sparing

## Abstract

**Background:**

To present the 18 months results from a prospective multicenter phase II randomized trial of short vs protracted urethra‐sparing stereotactic body radiotherapy (SBRT) for localized prostate cancer (PCa).

**Methods:**

Between 2012 and 2015, a total of 170 PCa patients were randomized to 36.25 Gy in 5 fractions (6.5 Gy × 5 to the urethra) delivered either every other day (EOD, arm A, n = 84) or once a week (QW, arm B, n = 86). Genitourinary (GU) and gastrointestinal (GI) toxicity (CTCAE v4.0 scale), IPSS, and QoL scores were assessed at baseline, at the 5th fraction (5fx), 12th weeks (12W), and every 6 months after SBRT. The primary endpoint was biochemical control at 18 months and grade ≥ 3 toxicity (including grade ≥ 2 for urinary obstruction/retention) during the first 3 months.

**Results:**

Among the 165 patients analyzed, the toxicity stopping rule was never activated during the acute phase. Maximum acute grade 2 GU toxicity rates at 5fx were 17% and 19% for arms A and B, respectively, with only 2 cases of grade 2 GI toxicity at 5fx in arm A. At month 18, grade ≥ 2 GU and GI toxicity decreased below 5% and 2% for both arms. No changes in EORTC QLQ‐PR25 scores for GU, GI, and sexual domains were observed in both arms between baseline and month 18. Four biochemical failures were observed, 2 in each arm, rejecting the null hypothesis of an unfavorable response rate ≤ 85% in favor of an acceptable ≥ 95% rate.

**Conclusions:**

At 18 months, urethra‐sparing SBRT showed a low toxicity profile, with minimal impact on QoL and favorable biochemical control rates, regardless of overall treatment time (EOD vs QW).

## Graphical abstract

Dose per fraction and overall treatment time can impact tolerance of prostate SBRT. Urethra‐sparing SBRT showed a low toxicity profile and minimal impact on QoL. An EOD or QW SBRT schedule had a comparable tolerance and efficacy at 18 months.

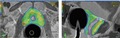

## 1 INTRODUCTION

Based on radiobiological estimates suggesting a low α/β ratio for prostate cancer treated with standard or moderate hypofractionated radiotherapy (RT),^1^ the question, if the linear‐quadratic (LQ) model still holds in scenarios in which extreme hypofractionation (≥6 Gy/fraction) is given, has been and still is a challenging one. Indeed, extreme hypofractionation delivered with stereotactic body radiotherapy (SBRT) has presently become an emerging and promising treatment modality for localized prostate cancer.^2^, ^3^, ^4^

Several extreme hypofractionated schedules have been reported so far differing in fractionation as well as in overall treatment time (OTT). Most frequently, 5 fractions of 7 or 7.25 Gy have been delivered for an assumed total equivalent dose to the tumor of 90 Gy in 2 Gy/fraction and a 5‐year biochemical relapse‐free (bRFS) survival rates exceeding 90%.^5^, ^6^ Results from the Scandinavian HYPO‐RT‐PC phase III trial have recently confirmed the noninferiority of extreme hypofractionation vs standard fractionated RT in terms of bRFS and late toxicity.^7^

Although bRFS rates from SBRT series are encouraging and can be considered a valid treatment option for prostate cancer patients, changes in dose per fraction and OTT have shown to impact treatment tolerance.^8^, ^9^ Indeed, acute bowel and urinary quality of life (QoL) are worse in patients treated every‐other‐day (EOD) compared with once‐weekly (QW) schedules.^9^ Nevertheless, the impact of OTT in terms of bRFS is not so well known and remains an open question when treating patients with SBRT.

The moderate toxicity reported with extreme hypofractionation should not limit, however, the effort to further reduce the dose to the organs at risk. It is well known from brachytherapy and standard fractionated external 3D or intensity‐modulated RT that urethra‐sparing techniques limiting the dose to the urethra and the bladder neck may be able to minimize urinary symptoms.^10^, ^11^ Based on the above assumptions we designed a prospective multicenter phase II randomized trial of SBRT delivered either EOD or QW exploring the potential role of OTT and of urethra sparing. In this study we report the first 18 months results of this trial.

## 2 METHODS AND MATERIALS

### 2.1 Patient characteristics

From August 2012 to December 2015, 170 prostate cancer patients were recruited in 9 centers in this prospective, multicenter phase II randomized trial (XX Trial). Inclusion criteria included patients of any age with a WHO performance status ≤ 2 and with a histologically confirmed adenocarcinoma of the prostate of Gleason Score ≤ 7, of tumor stage T1c‐3a, N0, M0, and an estimated risk of nodal involvement ≤ 20%.^12^ Previous transurethral prostate resection was allowed provided there was at least 8 weeks interval with SBRT. Patients with an International Prostate Symptom Score (IPSS) >19 were excluded.^13^ All patients were staged with multiparametric magnetic resonance imaging (mpMRI) and a bone scan in case of Gleason Score > 7 and PSA > 10 ng/mL.

Written informed consent according to ICH/GCP regulations was provided by all the patients before registration and prior to any trial‐specific procedures. The study was approved by the local ethical committee of every center. The study is registered at Clinical.Trials.gov (NCT01764646).

### 2.2 Treatment characteristics

Patients were randomly allocated (1:1) via a web‐based platform to receive the following target SBRT dose delivered QW or EOD: 36.25 Gy in 5 fractions to the whole prostate ± the seminal vesicles (SV) and 32.5 Gy in 5 five fractions to the urethra planning‐risk volume (uPRV), resulting in a biologically equivalent dose in 2 Gy fractions (EQD2) of approximately 90 Gy to the planning target volume (PTV) and 74 Gy to the uPRV (*α*/*β* = 1.5). With a *α*/*β* of 3 Gy for late toxicity, the corresponding EQD2 was 74 Gy and 62 Gy for the PTV and the uPRV, respectively ([Link], [Link]).^14^

Before the SBRT treatment, all patients have been implanted under ultrasound guidance with intraprostatic fiducial markers. Patients were simulated and treated with an empty rectum and full bladder. In 7 of the 9 centers patients were treated using an endorectal balloon (ERB) (Qlrad, Zwolle, the Netherlands). The PTV included the prostate ± the SV (cutoff threshold of 15%^15^) plus a 5‐mm expansion in all directions except posteriorly (3 mm). The uPRV was defined on CT images by contouring a 12 *French Foley* catheter inserted during the simulation only with a 3‐mm isotropic rim expansion and using mpMRI sequences to take into account possible variations in urethra position. Rigid coregistration with MRI undertaken under the same planning conditions was used for contouring purposes. Organs at risk were contoured according to RTOG guidelines^16^ and included the bladder wall and the rectal wall (defined as a 5‐mm and 3‐mm internal margin created from the external surface, respectively), the penile bulb, and the proximal femurs.

All patients were treated with a *Novalis *system (BrainLab AG and Varian Medical System) integrating a 6 degrees of freedom couch and an *ExacTrac* repositioning system. Treatments were delivered with either intensity‐modulated RT (n = 53) or volumetric‐modulated arc therapy (n = 112) techniques. Daily orthogonal images (kV or MV) with or without cone beam CTs were used to identify the implanted fiducials for image guidance. In accordance with the ICRU (*International Commission on Radiation Units and Measurements*) report 8, the plan normalization goal aimed to achieve 98% of the PTV receiving 95% of the prescribed dose (D_98%_ = 34.4 Gy) with a maximum of 2% of the PTV receiving no more than 107% of the prescribed dose (D_2%_ ≤ 38.8 Gy). Similarly, the goal for the uPRV was D_98%_ ≥ 30.9 Gy (95% of 32.5 Gy) and D_2%_ ≤ 34.7 Gy (107% of 32.5 Gy). Dose constraints for the rectal wall were V_36.25Gy_ < 5%, V_32.6Gy_ < 10%, and V_29Gy_ < 20%; for the bladder wall the constraints were V_36.25 Gy_ < 10%, V_32.6 Gy_ < 20%, and V_18.1 Gy_ < 50%; whereas for the femoral heads the constraint was D_2%_ ≤ 18.1 Gy.

Combined androgen deprivation therapy (ADT) with 6 months of LH‐RH agonists (2 months neoadjuvantly) was mandatory if 2 or more of the following tumor characteristics were present: ≥T2c, Gleason 4 + 3, PSA > 10 ng/mL, perineural invasion, and/or > 1/3 of positive biopsies.

### 2.3 Follow‐up

Patients were seen in a weekly basis during the treatment, at the 5th fraction, at week 12 since the start of SBRT, at months 6, 12, and 18 since randomization and yearly, thereafter. Toxicity was graded according to the Common Terminology Criteria for Adverse Events (CTCAE v4.03) grading scale, with acute toxicity considered as any adverse event occurring during the first 3 months. Medical management of treatment‐related toxicities was at the discretion of the treating physician. IPSS and QoL (EORTC QLQ‐C30 and QLQ‐PR25 questionnaires) assessments were also performed at the same endpoints. mpMRI, bone scan, choline‐, and/or PSMA‐PET were repeated in case of biochemical or clinical progression.

### 2.4 Statistical analysis

Primary endpoint of the study was bRFS at 18 months follow‐up calculated from time of inclusion until biochemical progression. Biochemical relapse was defined as a rise ≥ 2 ng/mL above the *nadir* PSA confirmed by a second observation taken 3‐4 weeks later (Phoenix definition). For sample size estimations, the single‐stage procedure of the designs proposed by Fleming was considered,^17^ as tabulated in Machin and Campbell.^18^ For a power of 90% and a significance level of 5%, a total of 76 patients had to be recruited in each treatment arm in order to detect a biochemical disease control rate of ≥ 95% against an undesirable level of ≤ 85% at 18 months of follow‐up (n = 165 patients for both arms considering a 8% rate of lost to follow‐up). Severe genitourinary (GU) and gastrointestinal (GI) acute toxicities during the first 3 months following the SBRT treatment were monitored during the whole study. A special attention was given to diarrheas, fecal incontinence, proctitis, rectal bleeding, and rectal pain for GI symptoms (grade ≥ 3), whereas for GU symptoms occurrence of hematuria, bladder spasms and pain (grade ≥ 3), and urinary retention and/or obstruction (grade ≥ 2) was recorded. As stopping rule, the procedure of Ivanova et al was applied, as implemented by the software quoted in the paper itself, for a significance level of 5% and a maximum tolerable rate of toxicity of 15%.^19^

Data description was performed using the mean, standard deviation, median, and interquartile range (IQR) for quantitative variables and percentages for qualitative ones. Efficacy analyses were performed on an intention‐to‐treat basis. QoL scores were described at each clinical surveillance follow‐up time by the mean, standard deviation, median, and range. All statistical analyses were performed with the statistical package Stata (StataCorp, 2009).

## 3 RESULTS

**Figure 1:**
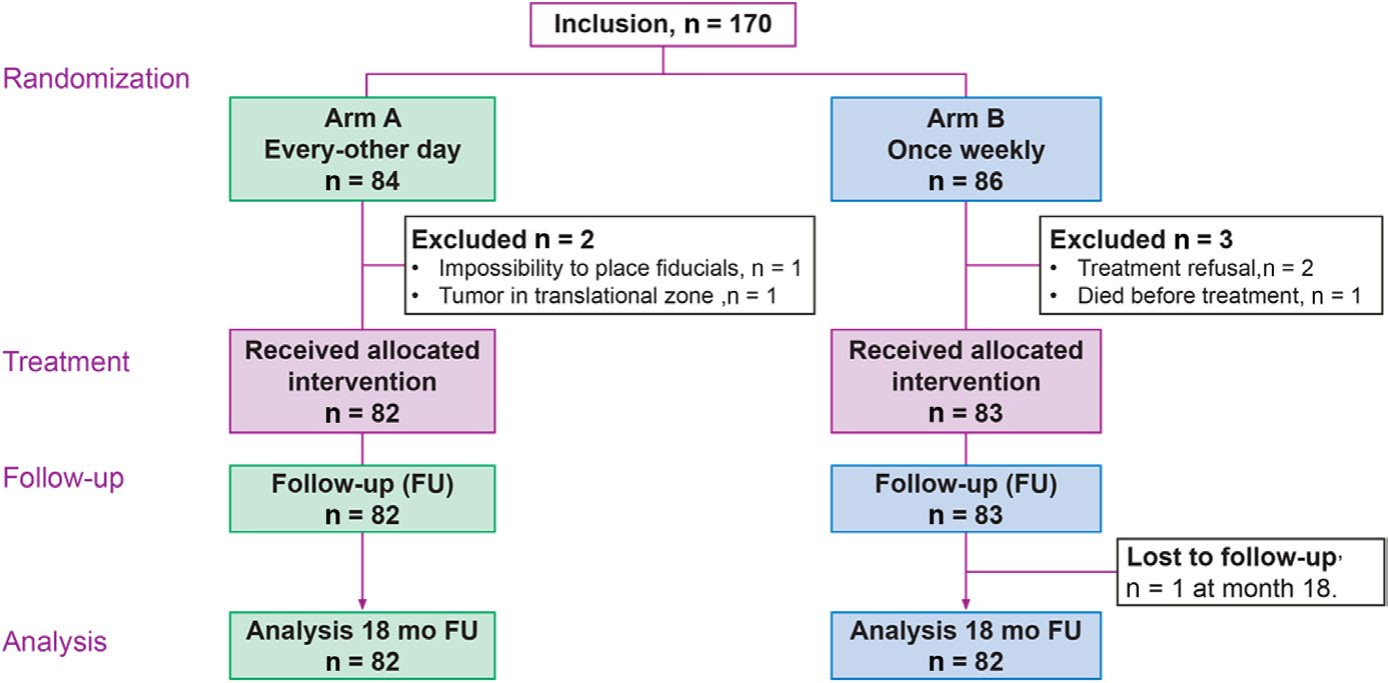
CONSORT (Consolidated Standards of Reporting Trials) diagram

A total of 170 men were found eligible and randomized in the study, with 165 (82 arm A and 83 arm B) treated and retained for the final analysis (Figure 1). Eighty‐two patients in each arm followed‐up to 18 months were evaluable for the primary endpoint and 18 months toxicity.

The majority of the patients were diagnosed with intermediate‐risk prostate cancer disease (63% and 64% for arms A and B, respectively). Patient demographic and clinical characteristics were well balanced between the 2 arms as illustrated on Table 1.

**Table 1:** Patient and tumors characteristics (n = 165)

Characteristics	Arm A	Arm B
Patients	82	83
Age (y)		
Median (range)	69 (51‐83)	70 (50‐82)
WHO performance status		
0	75	77
1	7	6
PSA at diagnosis (ng/mL)		
Median (range)	8.3 (2.7‐29)	7 (2.5‐29)
AJCC cT‐stage		
T1c	38	41
T2a	20	17
T2b	9	7
T2c	8	10
T3a	7	8
Gleason score		
3 + 3	32 (39)	32 (39)
3 + 4	32 (39)	36 (43)
4 + 3	18 (22)	15 (18)
NCCN risk group		
Low	18 (22)	18 (22)
Intermediate	52 (63)	53 (64)
High	12 (15)	12 (14)
SV involvement risk		
>15%	46 (56)	43 (52)
≤15%	36 (44)	40 (48)
ADT		
Yes	36 (44)	37 (45)
No	46 (56)	46 (55)

Abbreviations: ADT, androgen deprivation therapy; AJCC, American Joint Committee on Cancer; NCCN, National Comprehensive Cancer Network; SV, seminal vesicles; PSA, prostate‐specific antigen.

All patients completed the treatment schedule without interruptions. OTT deviated in 9 patients in arm A (OTT ranging from 10 to 12 days) and 2 patients in arm B (OTT = 30 days). One patient randomized in arm A was treated according to the arm B (OTT = 26 days).

### 3.1 Acute toxicity

**Figure 2:**
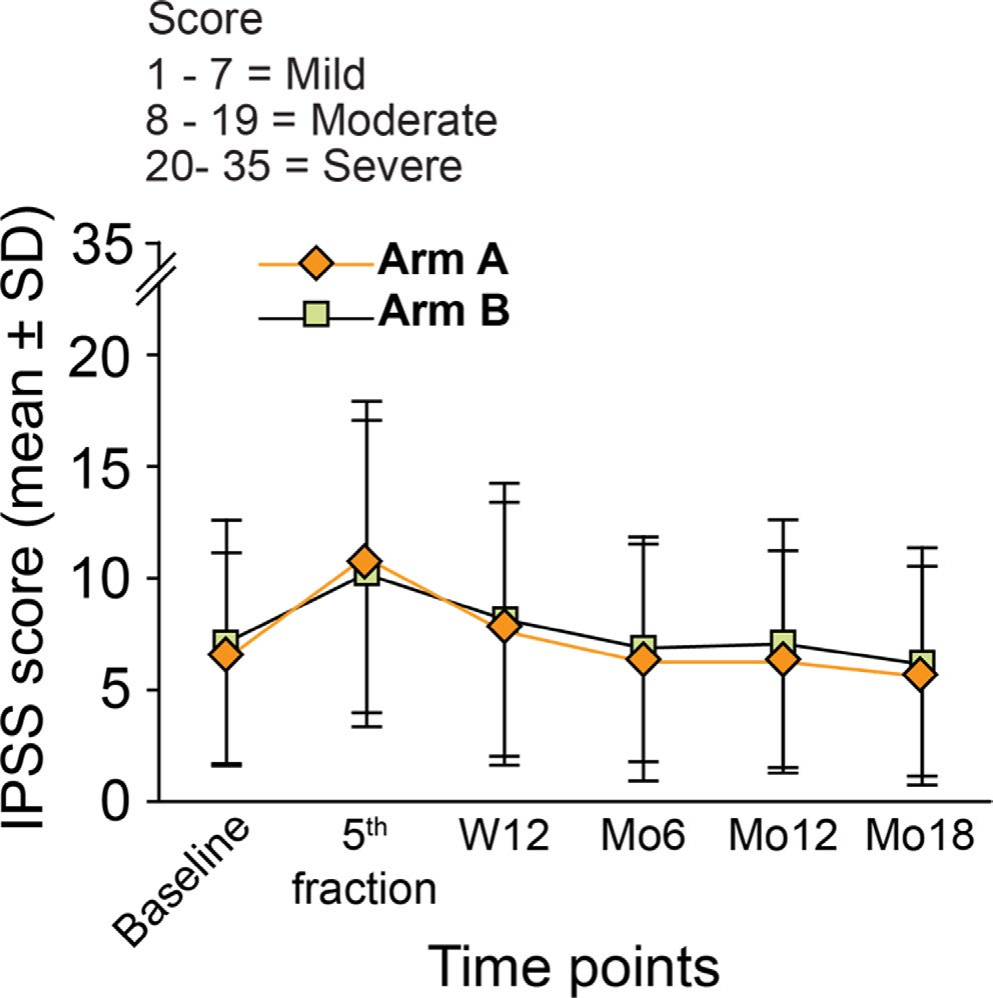
Mean ± SD values for IPSS scores for the first 18 months of follow‐up

In both arms, acute GU and GI toxicity was mild. The study stopping rule was never activated with only 1 case of grade 3 GU toxicity observed in arm B consisting of acute urinary retention requiring bladder catheterization. After the 5th fraction patients experienced a slight increase in grade 1 and 2 GU side effects consisting mainly in irritative and obstructive symptoms (bladder spasms, obstruction, cystitis, and urgency) declining at the next follow‐up control at week 12. The IPSS score increased from baseline from a mean value of 6.4 ± 5.5 and 7.1 ± 5.5 to 10.9 ± 7.0 and 10.2 ± 6.9 after the 5th fraction for arms A and B, respectively. The IPSS scores returned to the baseline at 3 months for both arms, with a mean value of 7.5 ± 5.9 and 8.1 ± 6.1, respectively (Figure 2). The percentage of patients presenting a satisfactory urinary QoL based on the IPSS score at baseline (scores 0‐2), at the 5th fraction and at the week 12 were 80%, 62%, and 78% for arm A and 77%, 67%, and 80% for arm B. GI toxicity remained mild with no Grade 3 events and < 2% of grade 2 side effects. GI side effects returned to baseline at 12 weeks and consisted mostly of grade 1 proctitis, mild rectal bleeding, diarrhea, and/or constipation. Acute maximum CTCAE v 4.0, GI, and GU toxicities are presented in Tables 2 and 3.

**Table 2:** First 18 mo CTCAE v4.0 gastrointestinal toxicities (n = 165)

	First 18 mo gastrointestinal grade ≥ 2 toxicities											
	Arm A						Arm B					
	Baseline (n = 82)	5fx (n = 82)	W12 (n = 82)	Mo6 (n = 80)	Mo12 (n = 82)	Mo18 (n = 79)	Baseline (n = 83)	5fx (n = 83)	W12 (n = 82)	Mo6 (n = 81)	Mo12 (n = 80)	Mo18 (n = 79)
Grade 0												
	65 (79%)	49 (60%)	67 (82%)	64 (80%)	59 (72%)	55 (70%)	63 (79%)	56 (68%)	60 (73%)	59 (73%)	59 (74%)	59 (74%)
Grade 1												
*Worst toxicity*	16 (20%)	31 (38%)	15 (18%)	16 (20%)	21 (26%)	22 (28%)	19 (20%)	27 (32%)	21 (26%)	22 (27%)	19 (24%)	18 (23%)
Grade 2												
*Worst toxicity* *	1 (1%)	2 (2%)	—	—	2 (2%)	2 (2%)	1 (1%)	—	1 (1%)	—	2 (2%)	3 (3%)
Diarrhea		1										
Constipation		1									1	1
Bleeding					1	2	1		1		1	2
Anal fissure	1				1							
Proctitis									1		1	1
Pain	1								1			
Grade 3												
Worst toxicity	—	—	—	—	—	—		—	—	—	—	—

Abbreviations: CTCAE = Common Terminology Criteria for Adverse Events v4.0; 5fx = 5th fraction; W12 = week 12.

* Some patients presented more than 1 grade 2 toxicity.

**Table 3:** First 18 mo CTCAE v4.0 genitourinary toxicities (n = 165)

	First 18 mo genitourinary grade ≥ 2 toxicities											
	Arm A						Arm B					
	Baseline (n = 82)	5fx (n = 82)	W12 (n = 82)	Mo6 (n = 80)	Mo12 (n = 82)	Mo18 (n = 79)	Baseline (n = 83)	5fx (n = 83)	W12 (n = 82)	Mo6 (n = 81)	Mo12 (n = 80)	Mo18 (n = 79)
Grade 0												
	49 (60%)	20 (24%)	41 (50%)	45 (56%)	46 (57%)	51 (65%)	59 (71%)	28 (34%)	39 (48%)	46 (57%)	47 (59%)	53 (67%)
Grade 1												
Worst toxicity	31 (38%)	48 (59%)	32 (39%)	26 (33%)	29 (36%)	24 (30%)	20 (24%)	39 (47%)	37 (45%)	30 (37%)	30 (37%)	23 (29%)
Grade 2												
Worst toxicity*	2 (2%)	14 (17%)	9 (11%)	9 (11%)	7 (9%)	3 (4%)	4 (5%)	16 (19%)	5 (6%)	5 (6%)	3 (4%)	3 (4%)
Cystitis		8	1	3	2	3	1	6	1	1	1	
Bladder spasm	1	1	2	2		1	1	3	1	2	1	3
Hematuria					1	1		1			1	
Incontinence			1				1	1	1	1		
Obstruction		5	2	1	3	1		5	2	2	1	
Pain		3			1	1		2		1		
Retention			1	2				1		1	1	
Urgency	1	4	2	1	1		2	3	2	1		1
Grade 3												
Worst toxicity*	—	—	—	—	—	1 (1%)	—	—	1 (1%)	—	—	—
Obstruction						1			1			

Abbreviations: CTCAE, Common Terminology Criteria for Adverse Events v4.0; 5fx, 5th fraction; W12, week 12.

* Some patients presented more than 1 grade ≥ 2 toxicity.

### 3.2 Six‐18 months late toxicity and biochemical control

Tables 2 and 3, display the late CTCAE v4.0 GI and GU maximum toxicity grades. Grade 2 GU toxicity decreased over time with < 4% of persistent toxicity at last follow‐up. Symptoms consisted mainly in cystitis and obstructive symptoms. Only 1 case of grade 3 GU toxicity (obstructive symptoms) was observed in arm A at month 18. GI toxicity was mild with up to 3% of grade 2 toxicity at 18 months follow‐up and no rectal toxicity observed in more than 70% of the patients in both arms. Among patients treated without ADT, grade 2‐3 erectile dysfunction was scored in 8.7% and 17.3% at baseline vs 18.6% and 15.9% at month 18 for arms A and B, respectively.

At the 6th, 12th, and 18th month of follow‐up, IPSS scores remained stable among the 2 arms, as illustrated in Figure 2. Mean ± SD IPSS scores at month 18 were 5.6 ± 4.9 and 6.2 ± 5.1 in arms A and B, respectively; whereas overall urinary satisfaction increased over time from 80% and 77% to 90% and 88% at last follow‐up in arms A and B, respectively.

**Figure 3:**
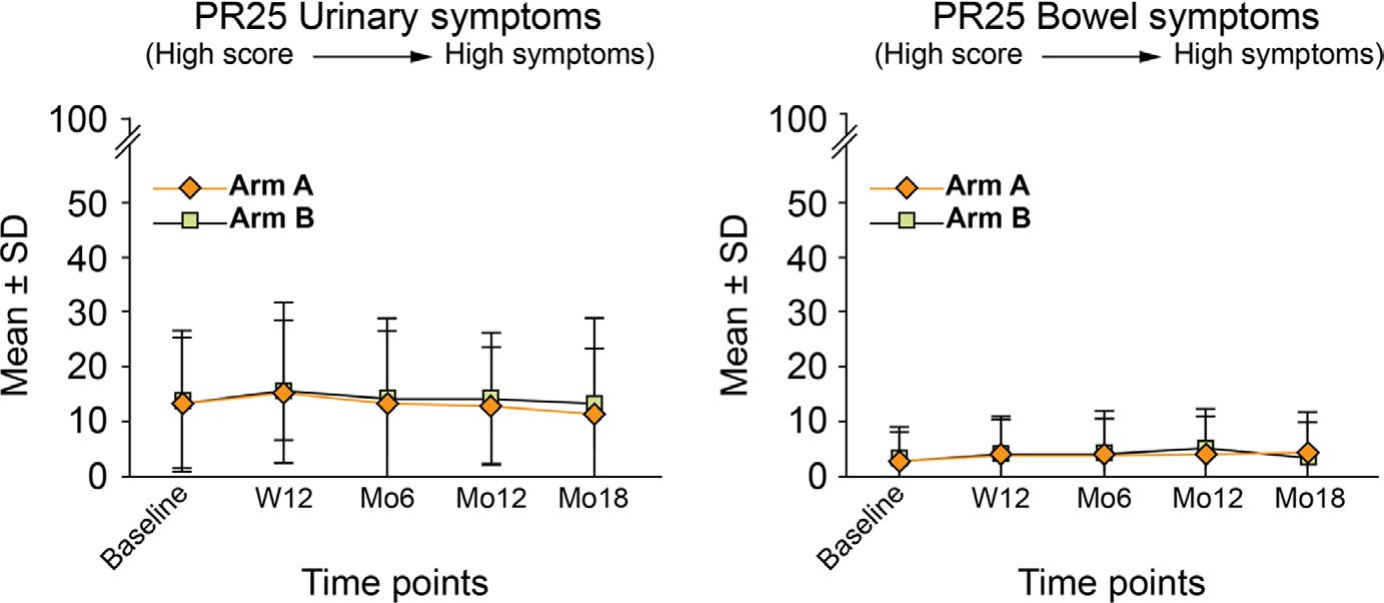
Mean ± SD values for EORTC QLQ‐PR25 scores for GU and GI domains for the first 18 months of follow‐up

No changes in EORTC QLQ‐PR25 scores for GU and GI domains were observed in both arms between baseline and week 12, while an improvement was observed at 18 months for the GU domain (median value of 12, IQR: 4‐16, and 8, IQR: 4‐16, at baseline, and month 18 in both arms) (Figure 3). Sexual domains (activity and functioning) remained comparable between the 2 arms and stable over the first 18 months of follow‐up on the whole patient population, as well as in a subgroup analysis of patients treated with exclusive SBRT without concomitant ADT ([Link], [Link]). The EORTC QLQ‐C30 global health status domain (Q29‐Q30) remained stable over time for both arms (median value of 83.3 at baseline and at month 18 in both arms).

Median PSA values decreased over time from a median value of 8.3 and 7.0 ng/mL (baseline) to 0.53 ng/mL and 0.46 ng/mL (at 18 months) in arms A and B, respectively (Figure SE). Twelve percent of patients experienced a biochemical progression (ie, PSA ≥ 0.2 ng/mL) between the 12th‐ and the 18th‐month follow‐up time points. Their PSA value was in all cases below the Phoenix definition for biochemical failure. At last follow‐up, 4 biochemical failures were observed, 2 in each arm, including an isolated biochemical relapse (PSA value of 8.1 ng/mL with negative PET‐CT imaging) and 3 patients with distant metastases. The null hypothesis of a biochemical control rate of at most 85% in favor of the alternative of a disease control rate in excess of 95% at 18 months was therefore rejected. No deaths were registered among the patients retained for the analysis.

## 4 DISCUSSION

In the last years, phase I to III trials of prostate SBRT have consistently reported excellent disease control rates with very mostly mild‐to‐moderate toxicity and minimal impact on patient's QoL.^6^, ^7^ Based on these studies, prostate SBRT can be considered nowadays as an appropriate definitive treatment modality for low‐ and intermediate‐risk prostate cancer. Nevertheless, the optimal SBRT dose, fractionation, and OTT remain open questions yet to be assessed, with a supposed superiority of SBRT over other treatment modalities needing further validation.

When this study was started back in 2012, few papers were available in the literature reporting on safety and efficacy matters of SBRT for prostate cancer and especially addressing matters like OTT. In a 2012 report from King et al, patients treated with 36.25 Gy in 5 fractions delivered EOD presented significantly lower low‐grade toxicity compared with patients treated on consecutive days, with an overall better late rectal QoL with the protracted schedule.^8^ With our trial, we aimed to test if extending the OTT to 28 days could further reduce toxicity of SBRT, while limiting the urinary toxicity with a urethra‐sparing technique.

We have been able to show a comparable toxicity profile of both treatment schedules, up to 18 months, with very few patients experiencing severe toxicity and with a minimal impact on QoL. These results are comparable to results from a pooled data analysis on 2142 prostate cancer patients showing a crude incidence of acute GU and GI toxicity of 9% and 3.3%, respectively.^6^ Of note, based on a systematic review of over 6000 patients treated on prospective studies with SBRT, 72% of prostate cancer patients were treated with SBRT delivered EOD, with only a minority of patients treated QW.^5^

So far, only one multicentric and prospective Canadian trial has reported on the impact of OTT on toxicity and QoL in patients treated with SBRT.^9^ Indeed, in the PATRIOT trial, Quon et al showed an improved acute bowel and urinary QoL by delivering prostate SBRT with a QW schedule compared with a shorter OTT using an EOD regimen. In their study, grade ≥ 2 acute GI toxicity using the Radiation Therapy Oncology Group grading scale was significantly higher in the EOD group (18.4%) compared to a 10.8% observed with the QW schedule, whereas no difference were observed in grade ≥ 2 acute GU toxicity (36.5% vs 32.9%). The different impact of OTT observed between the PATRIOT trial and our study may due to the following reasons: first, the SBRT dose delivered in the Canadian trial was higher, 40 Gy in 5 fractions compared to the 36.25 Gy in our study; second, in our study all patients were treated using a urethra‐sparing technique delivering a lesser dose (ie, 32.5 Gy/5fraction, equivalent to 62 Gy in 2 Gy/fraction, *α*/*β* = 3 Gy) to the urethra and bladder neck. Indeed, by extrapolating from external beam RT and brachytherapy series,^10^, ^11^ any dose optimization to the urethra and bladder neck may represent an appealing technique to reduce acute and long‐term GU toxicity and may explain the lack of differences in GU toxicity and urinary QoL between patients treated EOD or QW. Of note, a clear relationship between the delivered dose and the occurrence of late grade ≥ 3 GU toxicity has been demonstrated in a meta‐regression analysis of 5127 patients treated with prostate SBRT.^5^ This was confirmed by the low GU toxicity rates, comparable to our study, observed in 18 patients treated in a phase II SBRT trial delivering 37.5 Gy in 5 consecutive fractions to the prostate and between 33.2 and 35 Gy to the urethra.^20^

We do not know yet if long‐term disease control will not be negatively influenced by urethra sparing. In a randomized phase II trial including 16 patients with low‐risk prostate cancer, an urethra‐sparing technique failed to improve the urinary QoL while reporting a worse biochemical control compared with a standard whole prostate irradiation.^21^ In this study, the mean dose delivered to the proximal and distal urethra was 48.8 and 65.9 Gy, respectively. On the contrary, in our study all patients were treated with the same delivery technique using a homogenous dose optimization to the urethra,^14^ aiming to minimize GU toxicity while maintaining an acceptable tumor control to the possible microscopic periurethral disease (equivalent to 74 in 2 Gy per fraction, Figure SD). As far as rectal toxicity is concerned, in our trial the PTV margins were reduced posteriorly to 3 mm compared to the 5 mm used in the PATRIOT trial with the majority of patients treated with an inflated ERB. This approach helped to reduce the dose to the rectum by minimizing the irradiation of the postero‐lateral rectal wall^14^, ^22^ and by limiting intrafraction prostate motion,^23^ as well thus explaining the very low toxicity level observed in our study.

We have shown, in addition, a promising 18 months biochemical disease control > 95%, with only 4 biochemical failures, while 78% patients included in the trial presented with intermediate‐ or high‐risk disease. These results are comparable to the 96.9% biochemical disease control observed at 2 years in the prostate SBRT meta‐analysis from Jackson et al.^5^ At 18 months, and disregarding OTT, patients treated EOD or QW had the same median PSA values of 0.5 ng/mL, again, comparable to PSA levels previously reported in 5‐fraction SBRT studies.^24^ Of note, the median time to PSA nadir (median value, 0.2 ng/mL) was 40 months in a multi‐institutional series of 1062 patients treated with SBRT, with up to 26% of the patients experiencing a bounce at a median follow‐up time of 18 months.^24^ By keeping the duration of the QW schedule below 28 days we aimed to limit the negative impact of an accelerated repopulation of clonogenic cells, a phenomenon described when the OTT exceeds 4‐5 weeks.^25^

Several limitations of our study are to be acknowledged. We used an 18 months’ time‐point to assess the primary endpoint of bRFS, a much shorter follow‐up, than usual, to evaluate long‐term outcome. As stated before, this endpoint was decided at the study conception, when the LQ model reliability at high doses per fraction was uncertain^1^, ^26^ and only few SBRT series were available in literature, all reporting limited long‐term outcome. Moreover, with 18 months follow‐up, we were only able to assess acute, early late toxicity, and QoL. Nevertheless, regardless of the short follow‐up, we have been able to prove the safety and good tolerance during the first 18 months, a time‐point that can be considered robust to predict late toxicity events.^6^ Another possible drawback is the lack of an intermediate assessment of acute toxicity between the 5th fraction and week 12, considering that changes in GU and GI symptoms may be more evident during the weeks following the SBRT treatments. Last but not least, we acknowledge that by conducting two parallel phase II studies we limited any direct comparison between arms in terms of QoL, toxicity, and biochemical control.

Strengths of the present trial are the homogeneity of the treatment approach using the same delivery and IGRT technique for all patients, and a structured assessment and complete follow‐up protocol reporting physician‐scored and patient‐reported outcomes. Future and ongoing randomized trials comparing extreme hypofractionation regimens with either standard or moderate hypofractionation will help us to provide more insights on the role of SBRT for localized prostate cancer.

## 5 CONCLUSIONS

In the treatment of localized prostate cancer urethra‐sparing SBRT showed a very good toxicity profile, with minimal impact on QoL, and a promising biochemical control. At 18 months, tolerance and efficacy were comparable between SBRT delivered either EOD or QW. Nonetheless, a longer follow‐up is needed to assess the potential influence of OTT and urethra sparing on outcome and long‐term tolerance of such SBRT approach.

## CONFLICT OF INTEREST

The authors listed below report the following financial relationships: TZ reports research grant from Varian Medical Systems outside the submitted work; SJ reports no conflict of interest; SB reports no conflict of interest; AO reports no conflict of interest; CR reports no conflict of interest; AB reports personal fees from ViewRay Inc, outside the submitted work, and serves on the medical advisory board for ViewRay Inc; UA reports speaker fee from Varian and Brainlab, and has served in advisory boards for Brainlab and MSD; HM reports research grant from Philips Healthcare and research collaboration with MVision; ZS reports no conflict of interest; RM reports research grant from Varian Medical Systems and Brainlab.

## AUTHOR CONTRIBUTIONS

Thomas Zilli: Conceptualization, methodology, formal analysis, investigation, writing original draft, and writing review and editing. Sandra Jorcano: Investigation, writing original draft, and writing review and editing. Samuel Bral: Investigation, writing original draft, and writing review and editing. Carmen Rubio: Investigation, writing original draft, and writing review and editing. Anna Me Bruynzeel: Investigation, writing original draft, and writing review and editing. Angelo Oliveira: Investigation, writing original draft, and writing review and editing. Ufuk Abacioglu: Investigation, writing original draft, and writing review and editing. Heikki Minn: Investigation, writing original draft, and writing review and editing. Zvi Symon: Investigation, writing original draft, and writing review and editing. Raymond Miralbell: Conceptualization, methodology, formal analysis, funding acquisition, writing original draft, writing review and editing, and project administration. All authors approved the final version of the article prior to submission.

## Supporting information

Figure SA

Figure SB

Figure SC

Figure SD

Figure SE

## Data Availability

The datasets generated during and/or analyzed during this study are available from the corresponding author on reasonable request.
