# Supplementary figures and images for: Once‐a‐week or every‐other‐day urethra‐sparing prostate cancer stereotactic body radiotherapy, a randomized phase II trial: 18 months follow‐up results
Source: Cancer Med. 2020 Mar 11;9(9):3097–106. doi: 10.1002/cam4.2966 (PMC7196054; doi:10.1002/cam4.2966)

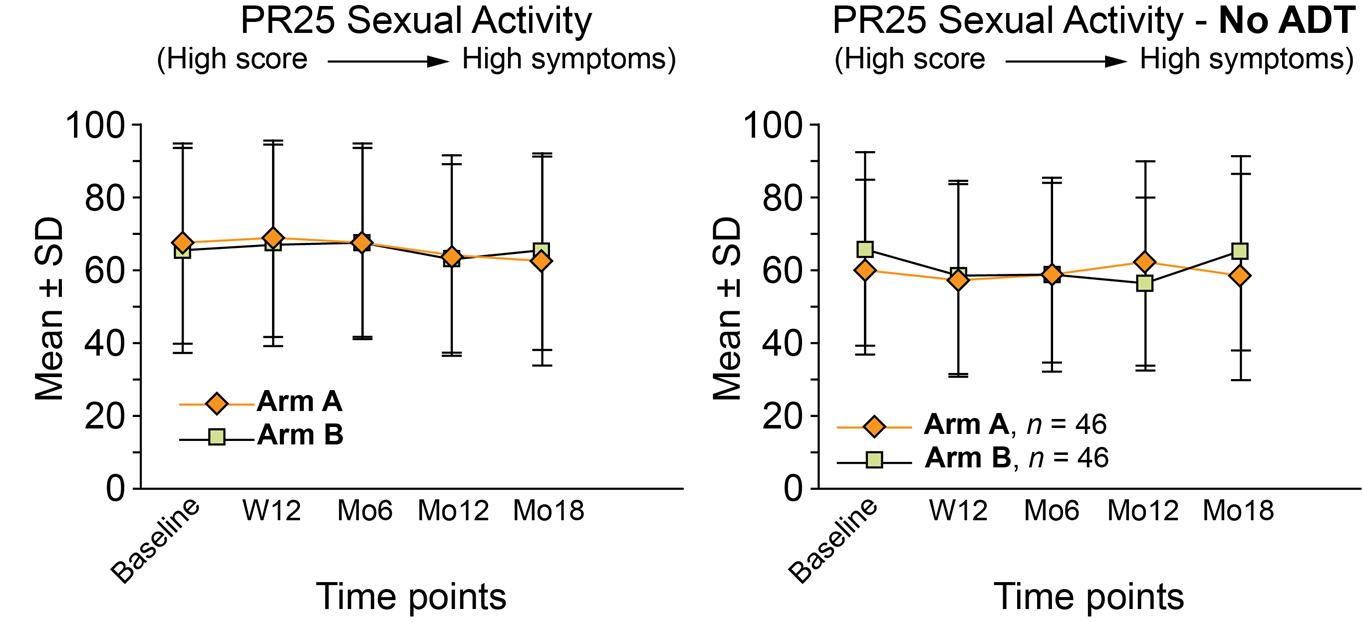

Supplement: Supplementary file 1 — Figure SA [file CAM4-9-3097-s001.tif]

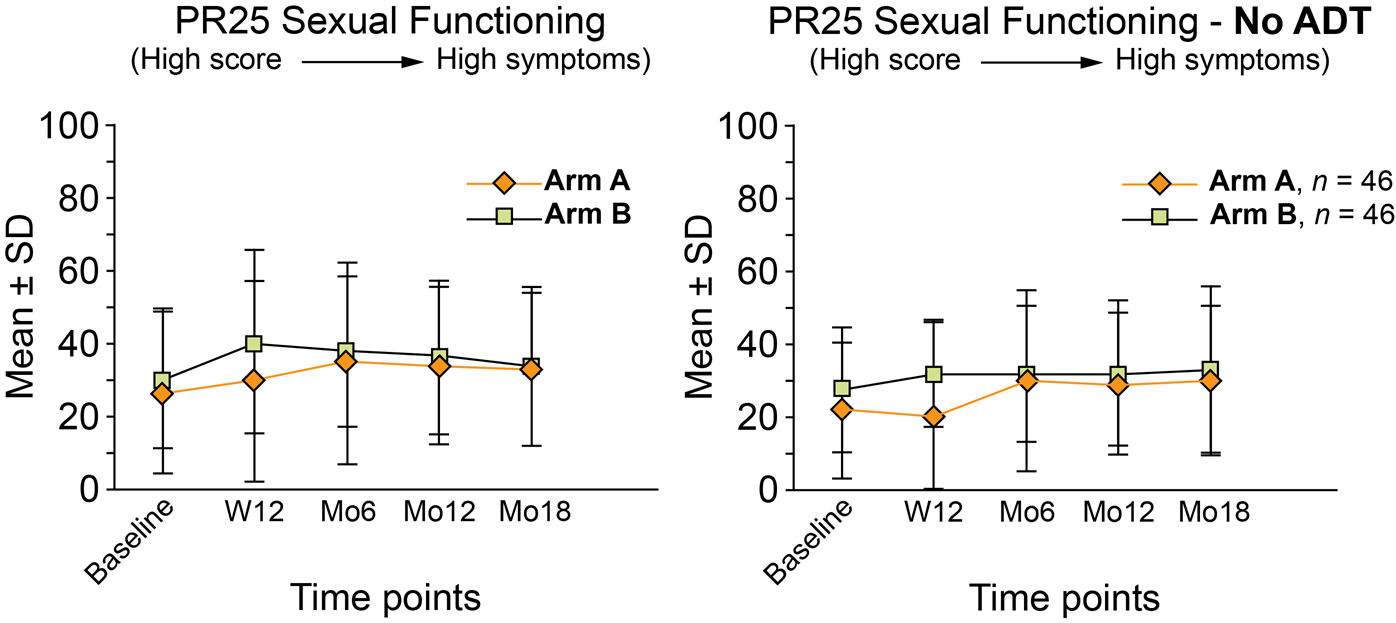

Supplement: Supplementary file 2 — Figure SB [file CAM4-9-3097-s002.tif]

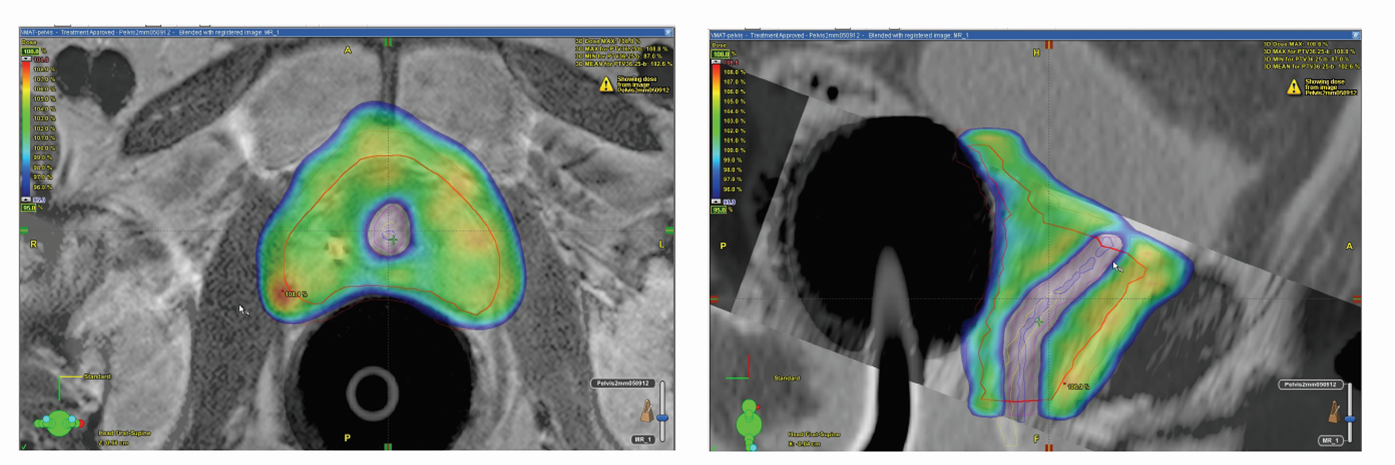

Supplement: Supplementary file 3 — Figure SC [file CAM4-9-3097-s003.tif]

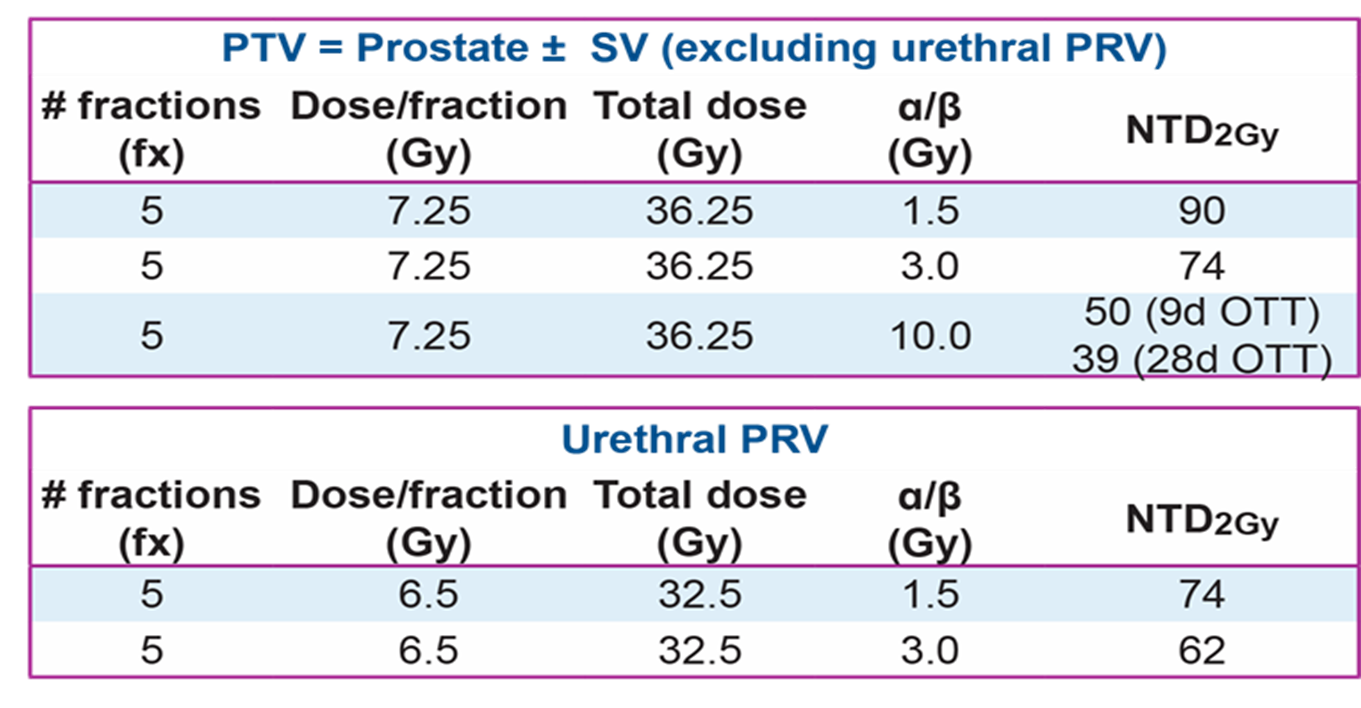

Supplement: Supplementary file 4 — Figure SD [file CAM4-9-3097-s004.tif]

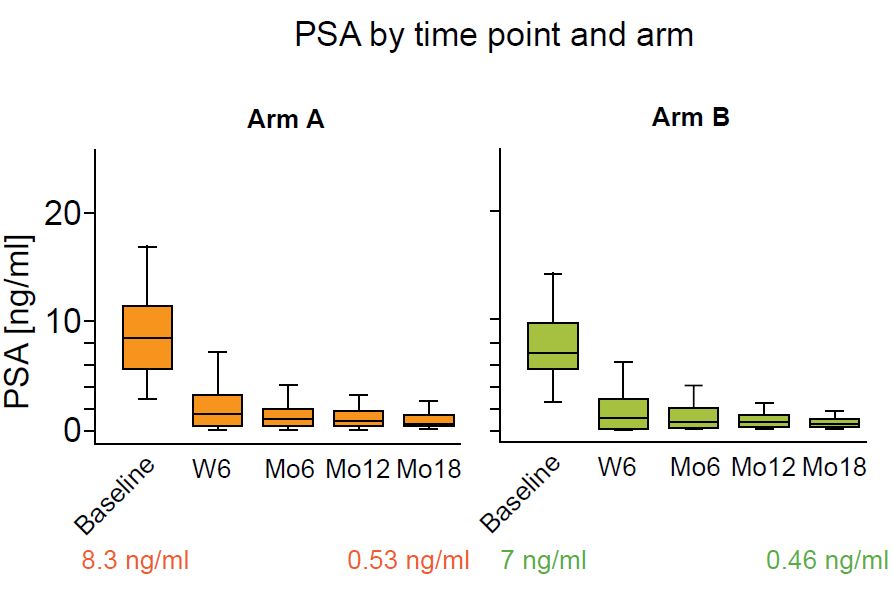

Supplement: Supplementary file 5 — Figure SE [file CAM4-9-3097-s005.tif]
